# Adrenal insufficiency causes life-threatening arrhythmia with prolongation of QT interval

**DOI:** 10.1007/s00380-015-0660-6

**Published:** 2015-03-15

**Authors:** Jin Komuro, Mitsunobu Kaneko, Kazutaka Ueda, Shuya Nitta, Masashi Kasao, Tetsuro Shirai

**Affiliations:** Department of Cardiology, Tokyo Metropolitan Police Hospital, 4-22-1 Nakano-ku, Nakano, Tokyo 164-8541 Japan

**Keywords:** Polymorphic ventricular tachycardia, Hypopituitarism, Long-QT syndrome, Adrenal crisis, Serum- and glucocorticoid-inducible kinase (SGK1)

## Abstract

A 63-year-old woman who had hypopituitarism was re-admitted to our hospital because of fever, diarrhea and disturbance of consciousness with life-threatening arrhythmia due to prolongation of the QT interval. She has been treated with hydrocortisone consequently, and has shown few ventricular arrhythmias with normalization of the QT interval. There have been several reports showing the case of prolonged QT interval with adrenal insufficiency, but there are few reports of isolated adrenocorticotropic hormone deficiency without any electrolytes imbalance that showed polymorphic ventricular tachycardia associated with QT prolongation. We discuss some possible mechanisms of how adrenal insufficiency causes life-threatening arrhythmia. Since lack of glucocorticoid hormone might induce prolongation of the QT interval, patients with adrenal insufficiency should be paid attention as candidates of lethal arrhythmias particularly when exposed to excessive stresses.

## Introduction

Adrenal crisis is a life-threatening emergency, however, the relation between adrenal crisis and life-threatening arrhythmia is not known. We describe a case with adrenal crisis and polymorphic ventricular tachycardia (VT).

## Case report

**Fig. 1:**
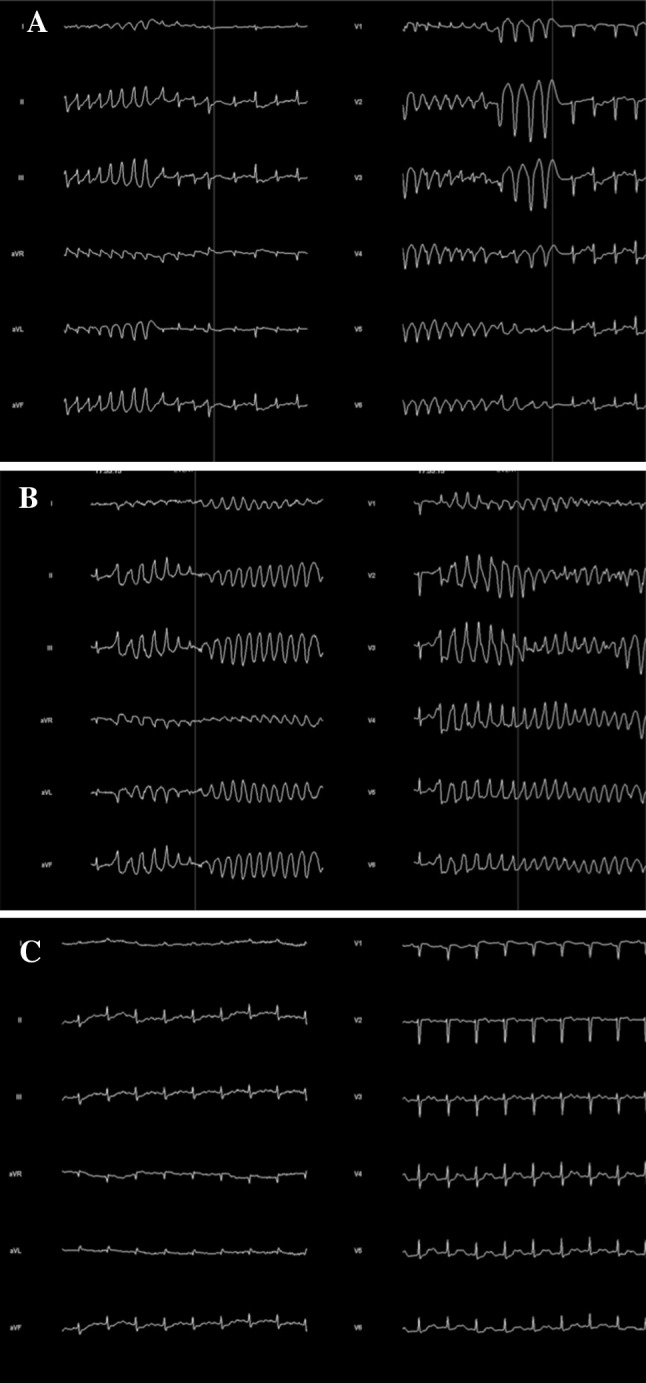
Polymorphic ventricular tachycardia recorded in 2009. **a** Short–long–short pattern of R–R cycles just before the occurrence of Torsades de Pointes. **b** Torsades de Pointes. **c** QT prolongation

**Fig. 2:**
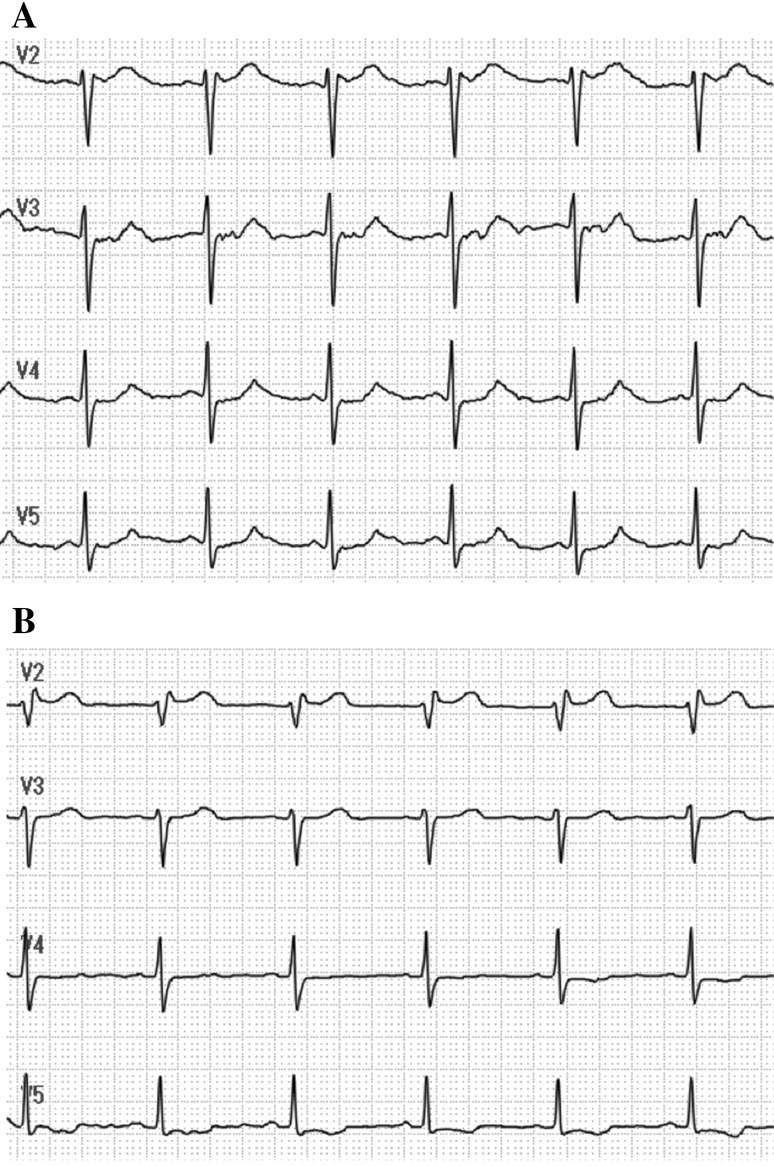
QT interval before and after administration of hydrocortisone. **a** Prolongation of the QT interval on admission. QTc, 521 ms. **b** Normalized QT interval after administration of hydrocortisone. QTc, 433 ms. QTc was measured at lead V5

A 63-year-old woman was re-admitted to our hospital because of fever, diarrhea and disturbance of consciousness with polymorphic VT. She had been first admitted to our hospital five years ago because of polymorphic VT (Fig. 1). Serum concentrations of cortisol, luteinizing hormone, follicle stimulating hormone, and prolactin had been very low with relatively low concentrations of corticotropin, while those of thyroid hormone and serum electrolytes levels had been almost normal. She had had a history of large bleeding by extrauterine pregnancy and her brain MRI had shown atrophy of pituitary, suggesting that her hypopituitarism was due to Sheehan’s syndrome [1]. She has no family history of VT and sudden deaths. On admission of this time, she had fever (38.0 ℃) with slightly lower blood pressure (92/60 mmHg). Her ECG showed prolongation of the QT interval (QTc 567 ms) (Fig. 2a) and ECG monitor recorded various arrhythmias such as sinus bradycardia, atrial flutter, paroxysmal atrial fibrillation, and polymorphic VT. Routine blood examination revealed normal levels of sodium (137 mEq/L), potassium (3.8 mEq/L), and calcium (9.9 mg/dl), and slightly high levels of magnesium (2.75 mg/dl). C-reactive protein and white blood cell counts were elevated 6.9 mg/dl and 11 × 10^3 ^μl, respectively. The rapid test of influenza type A was positive. Ultrasound echocardiography (UCG) showed normal cardiac size and function of the left ventricle. We intravenously administered hydrocortisone with large volume of physiological saline and started rapid ventricular pacing at the rate of 120 bpm. In the second day of the treatments, arrhythmias were not observed and the QT interval was normalized (QTc 394 ms) (Fig. 2b).

Although she has been treated with hydrocortisone for secondary adrenal insufficiency due to hypopituitarism, adrenal crisis might be induced by infection of influenza. When she arrived at our hospital, the QT interval was remarkably prolonged, however, it became normal within 12 h with replacement of glucocorticoids.

## Discussion

It is well known that hypothyroidism causes QT prolongation [2], but her serum concentrations of thyroid hormone had been almost normal from the first visit. On the other hand, her serum concentration of cortisol was very low (0.4 μg/dl) just before hospitalization and replacement of glucocorticoids promptly normalized the QT interval, suggesting that prolongation of the QT interval is due to lack of glucocorticoid hormone. There have been several reports showing the cases of prolonged QT interval with adrenal insufficiency. Previous reports described cases of Torsade de Pointes associated with hypopituitarism which were treated with steroid and thyroid hormone [6–8]. Other reports described cases of Torsade de Pointes associated with hypopituitarism and electrolytes imbalance [7, 8]. A case of adrenal insufficiency with severe hypokinesis of left ventricle and T wave inversion at ECG was also reported [7]. There are few reports of isolated adrenocorticotropic hormone deficiency without any electrolytes imbalance that showed polymorphic VT associated with QT prolongation [3]. In our case, the abnormality was only concentrations of cortisol and there were no electrolytes imbalance, hypothyroidism, or hypokinesis of the left ventricle. Although precise mechanisms of QT prolongation by glucocorticoid insufficiency are not known, there are some possibilities. Glucocorticoid has been reported to be important for the maintenance of membrane calcium transport function in the cardiac sarcoplasmic reticulum [3]. It has been also reported that glucocorticoid up-regulates expression levels of various ion channels, including I_Ks_ (mink, KvLQT1), and I_Kr_ (hERG, MiRP1) by inducing expressions of the serum- and glucocorticoid-inducible kinase (SGK1) [4]. Since these I_K _channels induce outward potassium currents, lack of glucocorticoid may extend duration of action potentials by reducing expressions of SGK1 and these ion channels [5]. In fact, there is a report showing that mutations in the hERG gene cause congenital long-QT syndrome [9].

Long-QT syndrome causes life-threatening ventricular arrhythmias such as Torsades de Pointes. Since lack of glucocorticoid hormone might induce prolongation of the QT interval, patients with adrenal insufficiency should be paid attention as candidates of lethal arrhythmias particularly when exposed to excessive stresses.

This study has been performed in accordance with the ethical standards laid down in the 1964 Declaration of Helsinki and its later amendments. We have obtained the informed consent from the patient.
